# Enhancing Clinical Decision Support with Adaptive Iterative Self-Query Retrieval for Retrieval-Augmented Large Language Models

**DOI:** 10.3390/bioengineering12080895

**Published:** 2025-08-21

**Authors:** Srinivasagam Prabha, Cesar A. Gomez-Cabello, Syed Ali Haider, Ariana Genovese, Maissa Trabilsy, Nadia G. Wood, Sanjay Bagaria, Cui Tao, Antonio J. Forte

**Affiliations:** 1 Division of Plastic Surgery, Mayo Clinic, 4500 San Pablo Road, Jacksonville, FL 32224, USA; 2 Department of Radiology AI IT, Mayo Clinic, 200 First St. SW, Rochester, MN 55905, USA; 3 Division of Surgical Oncology, Mayo Clinic, 4500 San Pablo Road, Jacksonville, FL 32224, USA; 4 Department of AI and Informatics, Mayo Clinic, 4500 San Pablo Road, Jacksonville, FL 32224, USA; 5 Center for Digital Health, Mayo Clinic, 200 First St. SW, Rochester, MN 55905, USA

**Keywords:** self-query retrieval, retrieval-augmented generation, large language models, clinical decision support, decision support systems

## Abstract

Retrieval-Augmented Generation (RAG) offers a promising strategy to harness large language models (LLMs) for delivering up-to-date, accurate clinical guidance while reducing physicians’ cognitive burden, yet its effectiveness hinges on query clarity and structure. We propose an adaptive Self-Query Retrieval (SQR) framework that integrates three refinement modules—PICOT (Population, Intervention, Comparison, Outcome, Time), SPICE (Setting, Population, Intervention, Comparison, Evaluation), and Iterative Query Refinement (IQR)—to automatically restructure and iteratively enhance clinical questions until they meet predefined retrieval-quality thresholds. Implemented on Gemini-1.0 Pro, we benchmarked SQR using thirty postoperative rhinoplasty queries, evaluating responses for accuracy and relevance on a three-point Likert scale and for retrieval quality via precision, recall, and F1 score; statistical significance was assessed by one-way ANOVA with Tukey post-hoc testing. The full SQR pipeline achieved 87% accuracy (Likert 2.4 ± 0.7) and 100% relevance (Likert 3.0 ± 0.0), significantly outperforming a non-refined RAG baseline (50% accuracy, 80% relevance; *p* < 0.01 and *p* = 0.03). Precision, recall, and F1 rose from 0.17, 0.39 and 0.24 to 0.53, 1.00, and 0.70, respectively, while PICOT-only and SPICE-only variants yielded intermediate improvements. These findings demonstrate that automated structuring and iterative enhancement of queries via SQR substantially elevate LLM-based clinical decision support, and its model-agnostic architecture enables rapid adaptation across specialties, data sources, and LLM platforms.

## 1. Introduction

The modern healthcare system is experiencing an unprecedented increase in demand due to the expanding aging population, the incidence and prevalence of chronic and infectious diseases, the emergence of new conditions, and the demand for elective procedures [1]. With the recent COVID-19 pandemic, worldwide medical systems noticed their lack of preparedness for providing prompt care when needed [2]. Moreover, the constant changes in evidence-based practices, combined with the heavy workload physicians face, can lead to burnout and increase the risk of errors, such as misdiagnoses or treatment mistakes [3]. Artificial Intelligence (AI) presents a promising solution, as it mimics human reasoning, judgment, and behavior and can easily handle the increasing volume of medical data, aiding physicians in accessing and maintaining updated knowledge [1,4,5,6,7]. Advances in computing power and the growing volume of electronic health record (EHR) data have made AI a practical tool in healthcare [8]. Estimates suggest that AI could boost labor productivity by 11–37% by 2035 and add $17.7 trillion to the global economy by 2030 [4]. By improving diagnostic accuracy, streamlining physician workflows, reducing errors, and supporting patient monitoring, AI can help meet rising healthcare demands in a more cost-effective way [9,10].

A notable area of AI in medicine involves Large Language Models (LLMs), Natural Language Processing (NLP) models designed to comprehend and generate human language [11,12]. By rapidly curating and synthesizing relevant evidence, LLMs can mitigate the medical information overload [13,14]. Recent studies have demonstrated the capabilities of these models in providing nuanced guidance for appropriate treatment decisions, showcasing their potential for clinical decision support [15,16,17,18,19,20,21]. However, LLMs are not without limitations, as their code to generate responses based on next-word predictions sometimes precedes factuality, leading to the generation of fabricated responses when lacking information, e.g., hallucination, which can result in deviations from established medical practices [12,22,23]. Hallucinations can be presented as subtle errors and stated in a convincing manner that makes users believe in their veracity, and this is especially dangerous in clinical decision-making [22]. In a systematic review analyzing the ethical considerations of LLM applications in surgery, the accuracy of the models and their content was identified as the most frequently cited concern, demonstrating the significant weight this obstacle carries for proper implementation into clinical settings [23]. Even when baseline domain knowledge appears strong on medical benchmarks, recent work shows that clinically irrelevant properties, such as typos, slang, or writing style, can sway LLM treatment recommendations, revealing brittleness not captured by standard exams [24]. These observations make explicit evaluation and reporting standards essential in healthcare applications [25,26].

Fine-tuning, prompt engineering, in-context learning (ICL), and retrieval-augmented generation (RAG) are techniques implemented to address these limitations [27,28,29,30]. RAG enables LLMs to access up-to-date, external, and validated sources of information that can be tailored to their specific use cases. This ensures that the information retrieved from the models is accurate and controlled, therefore increasing LLMs’ safety, reliability, explainability, and accountability [28,30,31,32,33,34,35]. Compared to fine-tuning, RAG has proven to be superior in enhancing the models’ accuracy and reducing hallucinations [36,37,38,39]. Additionally, LLMs become more flexible and adaptable to new knowledge while reducing resource intensity, as the models do not need constant and extensive retraining [37,38]. Recent studies have demonstrated the potential of RAG-LLMs for enhancing clinical decision support in different areas, including nephrology, surgery, and oncology [31,32,40,41]. Despite these advantages, RAG models are limited by the complexity of optimizing the functionality of the components in their architecture [28]. A recent systematic review of point-of-care QA systems found that most evaluations still use unrealistically simple questions, rarely communicate uncertainty, and often lack user studies, highlighting a gap between research prototypes and clinical needs [42]. This is not different when it comes to RAG; performance is sensitive to retrieval design choices, and evaluation is not yet standardized [43].

In specialized areas such as medicine, the inherent semantic gap between natural language user queries and the document structure within the knowledge database may limit the effectiveness of RAG models in retrieving the most pertinent information [44,45,46]. This may be caused by the input query not fully capturing the specific knowledge required for retrieval, leading to less relevant documents being retrieved and ultimately poor performance from the LLM [44,46]. Therefore, metadata-aware retrieval can be critical in clinical contexts, as naïve RAG pipelines often struggle when document metadata, such as date, patient population, or study design, must guide the selection of the most relevant evidence [47,48]. Self-query retrieval (SQR) addresses this gap by having the LLM generate follow-up, structured queries by extracting metadata elements from the initial user question to filter and retrieve precisely targeted documents. In a typical clinical scenario, where time is essential, offloading the prompt-structuring task to the model minimizes cognitive and workflow burden on physicians.

In this work, we propose a self-query retrieval framework in which the RAG-LLM automatically reformulates free-text clinical questions into established clinically oriented schemas, such as PICOT (population, intervention, comparison, outcome, time), SPICE (setting, population, intervention, comparison, evaluation), or specialty-specific frameworks, and then uses these elements to guide document retrieval [49,50]. To further optimize retrieval, we integrate iterative query refinement (IQR), which allows the model to dynamically adjust its search terms in response to initial results and user feedback until it meets an established quality score [44,45,51,52]. This approach ensures that retrieved information aligns closely with clinicians’ needs, enhancing both the precision and explainability of LLM-generated recommendations. Additional work has found that follow-up questions and adaptive retrieval improve the quality of the answers on knowledge-intensive tasks, strengthening the case for structured, metadata-aware querying for clinical uses [53].

This paper is the first one to present advanced techniques for developing a standardized, generalizable RAG framework for clinical decision support. Here, we detail our self-query retrieval and iterative refinement methods and demonstrate how they improve accuracy, relevance, and information retrieval performance metrics in a specific clinical setting addressing patient questions post-rhinoplasty.

Contributions:

(a) Adaptive Self-Query Retrieval: Clinically oriented schemas for metadata-aware retrieval are implemented, with a robust fallback mechanism when reliable auto-population is not feasible.
(b) Iterative Query Refinement: Implementation of a self-critiquing/rewriting loop that updated the structured queries and related data until a predefined quality criterion was met.
(c) Composite context scoring: Evidence is prioritized using a principled blend of semantic similarity, lexical overlap, and length normalization to mitigate chunk-length bias.
(d) Model-agnostic, modular design: LLM, embedders, and corpora are swappable without major architectural changes.
(e) Dual evaluation: Clinician-facing metrics (accuracy and relevance) are paired with information-retrieval metrics (precision, recall, and F1) to link clinical utility with retrieval quality.
(f) Empirical gains over a basic RAG baseline: Steady increase in all metrics evaluated from the basic RAG model to the complete pipeline implementing SQR with fallback mechanism and IQR.
(g) Statistical transparency: Standardized reporting in Results (two-sided α = 0.05; ANOVA with Tukey for accuracy/relevance; IR metrics descriptive with clear definitions).
(h) Clinically grounded proof-of-concept: Demonstrated on postoperative rhinoplasty queries, with a path to broader specialty-specific deployments.

## 2. Methods

**Figure 1:**
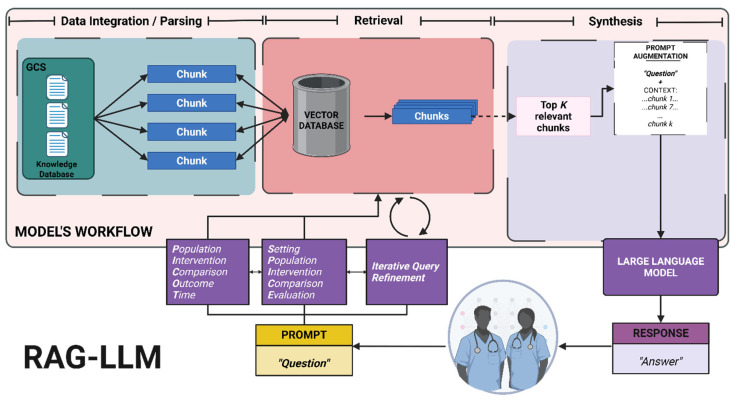
Implementation of our proposed self-query retrieval with iterative query refinement, using PICOT and SPICE frameworks for enhancing retrieval in RAG-LLM for clinical decision support [55].

As model performance is highly influenced by prompt quality, delegating this task to the model can substantially decrease clinicians’ burden while enhancing retrieval accuracy [54]. To date, a standardized prompting method tailored for RAG models has not been established. To address this challenge, we are proposing a self-query retrieval (SQR) framework that restructures prompts into high-quality, clinically oriented question frameworks and integrates an iterative query refinement (IQR) layer to dynamically adjust and enhance queries based on intermediate retrieval results and the model’s feedback. To test our pipeline, we utilized two different clinical question frameworks, PICOT (Population, Intervention, Comparison, Outcome, Time) and SPICE (Setting, Population, Intervention, Comparison, Evaluation), due to their proven performance [49,50]. However, because of the model’s agnostic nature, it can adopt alternative custom formats tailored to specific clinical settings. Figure 1 summarizes our proposed framework.

### 2.1. Study Design and Setting

We developed three RAG models, each leveraging SQR with a different prompt strategy. The first model restructured the prompts based on the PICOT format, while the second model restructured them using the SPICE framework. Conversely, the third model consisted of the complete pipeline, where queries were initially restructured using PICOT, reverted into SPICE whenever at least two PICOT components could not be determined, and incrementally adjusted using IQR. The three different model pipelines, along with a naive RAG model, were implemented on Gemini-1.0 Pro.

We developed a set of 30 queries addressing common postoperative concerns among rhinoplasty patients at our clinic, as well as potential complications that can arise following the procedure. Questions ranged from simple to complex. The model was provided with the type of procedure performed (e.g., open rhinoplasty) and the timing of the procedure (e.g., “1 week ago”). Each question was presented to the four models without employing any additional prompting engineering techniques. After every question was asked to one model, another was tested.

### 2.2. Pipeline Implementation

#### 2.2.1. Query Decomposition and Self-Query Generation

Upon receiving a free-text clinical question (denoted as Q), the first step is to instruct the LLM to reformulate that question into a structured PICOT string (Population, Intervention, Comparison, Outcome, Time). For example, given “I had open rhinoplasty two days ago. How should I manage my swelling and pain?”, the LLM is prompted to extract:

➔ Population: “patient at postoperative day 2 after open rhinoplasty,”
➔ Intervention: “swelling control and pain management,”
➔ Comparison: “N/A” (if not explicitly stated),
➔ Outcome: “reduced edema and adequate analgesia,”
➔ Time: “48 h post-op.”

This PICOT formulation becomes the self-query for retrieval, replacing the original raw text. In cases where the LLM cannot identify at least two PICOT components (i.e., if neither a Comparator nor an Outcome can be reliably extracted), the system automatically falls back to a SPICE schema. The SPICE-derived string, similarly, is used to guide retrieval. This two-stage approach ensures that our pipeline can handle both rigorously structured clinical inquiries and broader, less explicit questions, guaranteeing that the subsequent retrieval steps always receive a coherent prompt tailored for the specific clinical setting in place.

#### 2.2.2. Document Chunking via Recursive Character Splitting

For this initial testing, our knowledge corpus C consisted of authoritative sources on rhinoplasty. All documents in C are preprocessed through hierarchical chunking. Each document is first divided along paragraph boundaries; if a paragraph exceeds 1000 characters, it is further split at sentence breaks and, if necessary, at commas or semicolons until each fragment falls below the 1000-character threshold. A fixed overlap of 100 characters is maintained between adjacent chunks to preserve contextual continuity at chunk boundaries. This recursive character splitting (RCS) method ensures that no clinically relevant sentences are lost while keeping each chunk small enough for efficient embedding and retrieval.

#### 2.2.3. Embedding Index Construction

Once chunking is complete, we compute a dense-vector embedding for each chunk using the Vertex AI Embeddings service (e.g., the text-embedding-004 model). Each chunk $d_{i}$ is mapped to a vector $V_{d_{i}\;}\epsilon \;R^{D}$. At the same time, the structured query, either PICOT or SPICE, is flattened into a single “self-query” string, which is likewise embedded to produce $V_{Q\;}\epsilon \;R^{D}$. All chunk embeddings, along with relevant metadata tags (such as “postoperative day” or “rhinoplasty”), are stored in a Chroma vector index to enable efficient nearest-neighbor retrieval and metadata-based filtering during subsequent steps.

#### 2.2.4. Initial Retrieval and Composite Context Scoring

With the self-query embedding $V_{Q\;}$ in hand, we perform a cosine-similarity search against all chunk embeddings in the index. Specifically, for each chunk $d_{i}$, we compute Equation (1).

(1) $$Ssem(Q,d_{i})=cos(V_{Q\;},V_{d_{i}\;})=\frac{{(V}_{Q\;}\times \;V_{d_{i}\;})}{\left|V_{Q\;}\right|\;\left|V_{d_{i}\;}\right|},$$

where

- Q is the free-text clinical question;
- $d_{i}$ refers to the embedded chunks.

and retrieve the top-k chunks whose embeddings are most semantically similar to $V_{Q\;}$. To refine the candidate set, each of the top-k chunks is then scored with a composite retrieval function (Equation (2)).

(2) $$S(d_{i})=0.5\;Ssem(Q,d_{i})+0.3\;S_{lex}(Q,d_{i})+0.2\;S_{len}(d_{i}),$$

where

- $S_{lex}(Q,d_{i})$ is the lexical overlap, defined as the proportion of shared keywords between the flattened query and the chunk $d_{i}$.
- $S_{len}(d_{i})$ is a length-normalization term, equal to $min\;(\left|d_{i}\right|,\;1000)\;/\;1000$, which discourages overly long passages from dominating retrieval.

Through ablation testing, we found that assigning 50% weight to the semantic component prioritizes conceptual alignment with the query, 30% to lexical overlap anchors retrieval in precise, factual terms, and 20% to length normalization prevents chunk length bias. This weighting strikes an effective balance between relevance and conciseness, producing high-precision contexts for downstream synthesis.

#### 2.2.5. Initial Answer Generation and Scoring

The top-m chunks (commonly m=3) ranked by $S(d_{i})$ are concatenated, along with the flattened self-query, into a composite prompt for the Gemini 1.0-pro 002 LLM. This prompt is structured to ask the model, for instance, “Using the following retrieved passages (listed in order of relevance), please provide evidence-based postoperative guidance for the query. Cite which chunk supports each recommendation.” The LLM’s response, denoted $A^{0}$, is then evaluated for relevance and factual alignment. We compute a confidence score $s^{0}$ Via two methods: re-embedding $A^{0}$ to measure cosine similarity against $V_{Q\;}$ and/or each evidence embedding, and, when necessary, invoking a smaller cross-encoder to verify that each claim in $A^{0}$ is supported by at least one retrieved chunk. The resulting $s^{0}\in \left[0,1\right]$ quantifies how well the initial answer aligns with the evidence set.

#### 2.2.6. Adaptive, Corrective Self-Refinement Loop

In the adaptive, corrective self-refinement loop, the pipeline repeats retrieval and answer synthesis for up to ten iterations (Tₘₐₓ = 10), ensuring that each pass corrects previous errors and incrementally improves factual grounding. At the start of each cycle, the LLM is prompted to critique its prior response by identifying unsupported or ambiguous statements and suggesting necessary corrections. This feedback is then integrated into the original query Q to produce a refined query $Q^{t}$,  which adds domain-specific terms or clarifications that address gaps in the previous answer. Once $Q^{t}$  is formed, cosine similarities between its embedding and every document-chunk embedding are recomputed, and each chunk is re-ranked according to the composite score S = 0.5·Sₛₑₘ + 0.3·Sₗₑₓ + 0.2·Sₗₑₙ. The top-ranked chunks constitute an updated evidence set $D_{retrieved}^{t}$, which is then concatenated with the refined query to form a revised prompt. Gemini produces a new answer $A^{t}$,  in response to this prompt, and a corresponding confidence score sᵗ is calculated by measuring alignment with the retrieved context (e.g., Via cosine similarity or a claim-verification subroutine). Throughout all ten passes, the tuple (t,  $Q^{t}$,  $A^{t}$,  $s^{t}$) is logged, and upon completion of the tenth iteration, the answer with the highest confidence score is selected as the final output along with its supporting evidence. Empirical convergence testing has shown that, by iteration ten, both the composite retrieval score S and the phrasing of the refined query and answer exhibit negligible change; accordingly, imposing the ten-step cap balances iterative improvement against computational cost and latency.

#### 2.2.7. Fallback Mechanism and Final Answer Synthesis

Whenever the PICOT decomposition fails to extract at least two coherent components (e.g., Population, Outcome, or Time), the pipeline reverts to SPICE. Under SPICE, the LLM extracts Setting, Population, Intervention, Comparator, and Evaluation from the original question. This SPICE string then replaces PICOT as the self-query, and all subsequent steps (composite scoring, answer generation, and iterative refinement) proceed identically. By embedding this fallback logic, our system ensures robust handling of both granular, guideline-style clinical questions and broader patient inquiries.

Once convergence is reached or the iteration limit is met, we select the answer $A^{t}$ associated with the highest confidence score $s^{t}$. The final output is accompanied by citations to the specific chunks that support each recommendation, preserving transparency and enabling clinician review. All intermediate logs, including structured sub-queries, retrieved chunk IDs, composite scores, and LLM outputs, are retained for auditability and future analysis.

#### 2.2.8. Implementation Details

The entire pipeline is orchestrated in Python 3.10.18, utilizing the Vertex AI API for prompt-based decomposition and answer synthesis, as well as a Chroma index for vector storage and nearest-neighbor retrieval. Embeddings are generated via Vertex AI Embeddings (text-embedding-004), and cosine similarity calculations are performed with NumPy operations. Composite scoring and iterative logic are handled by a driver script that monitors convergence and enforces the ten-step cap.

### 2.3. Evaluation Tools and Outcome Metrics

To evaluate the medical accuracy of the models’ responses, we utilized a 3-point Likert scale, where 1 point represented an entirely wrong response that contradicted established medical knowledge, 2 points represented a somewhat accurate response that contained a mix of correct and incorrect information, and 3 points represented a completely accurate response that matched the information reference sources and known practice. Alternatively, to determine the relevance and value of the responses to each specific clinical query, we scored them on a 3-point Likert scale with the following values: 1 point, irrelevant, the answer did not provide helpful information; 2 points, somewhat relevant, the answer offered some general information but lacked specific guidance; 3 points, relevant, the answer directly addressed the clinical scenario and provided helpful, actionable steps. We used as ground truth authoritative sources on rhinoplasty, including “Essentials of Septorhinoplasty: Philosophy, Approaches, Techniques,” Postoperative Care and Management; “Plastic Surgery: A Practical Guide to Operative Care,” Rhinoplasty; “Rhinoplasty Cases and Techniques,” Postoperative Care; “Plastic Surgery, Volume 2: Aesthetic Surgery (Fifth Edition),” Open Rhinoplasty Technique, Closed Rhinoplasty Technique, and Secondary Rhinoplasty.

We evaluated the models’ information retrieval performance using precision, recall, and F1 score. Precision was defined as the fraction of retrieved items that were indeed relevant (Precision = TP/(TP + FP)), recall was the fraction of all relevant reference items actually retrieved by the model (Recall = TP/(TP + FN)), reflecting completeness of retrieval, and F1 score represented the harmonic mean of precision and recall to balance these two aspects (F1 = (2 × Precision × Recall)/(Precision + Recall)). We considered a true positive (TP) any passage or recommendation retrieved by the models that appears in the authoritative sources provided; a false positive (FP) any retrieved item not present in those sources or irrelevant for the clinical scenario; and a false negative (FN) any relevant item that the model failed to retrieve. Higher precision denoted fewer irrelevant results, higher recall represented fewer missed items, and a higher F1 score indicated an optimal trade-off between precision and recall.

### 2.4. Statistical Analyses

For medical accuracy and relevance, we calculated and recorded the mean, mode, standard deviation (SD), and range using a Microsoft Excel spreadsheet (Version 2503 Build 16.0.18623.20266 64-bit). To compare the models’ performance, we applied a one-way analysis of variance (ANOVA) and Tukey’s post hoc analysis for pairwise comparisons when applicable. Both tests were calculated using Microsoft Excel’s statistical package. A *p*-value of less than 0.05 was considered statistically significant. As information retrieval performance metrics primarily serve to illustrate practical retrieval improvements rather than assess inferential differences, we did not conduct formal hypothesis tests and reported only descriptive summary statistics.

## 3. Results

All experiments were conducted on 30 unique clinical queries addressing patient concerns following rhinoplasty. Unless stated otherwise, all hypothesis tests were two-sided with α = 0.05. Continuous outcomes are summarized as mean ± SD and categorical variables as *n* (%). Medical accuracy and relevance were compared across models using one-way ANOVA with Tukey’s post hoc tests for pairwise contrasts; we report group summaries with corresponding *p*-values. Information-retrieval metrics (precision, recall, and F1) are presented descriptively to illustrate retrieval behavior and were not subjected to formal hypothesis testing. Full analytic details are provided in Methods Section 2.4.

### 3.1. Medical Accuracy

**Figure 2:**
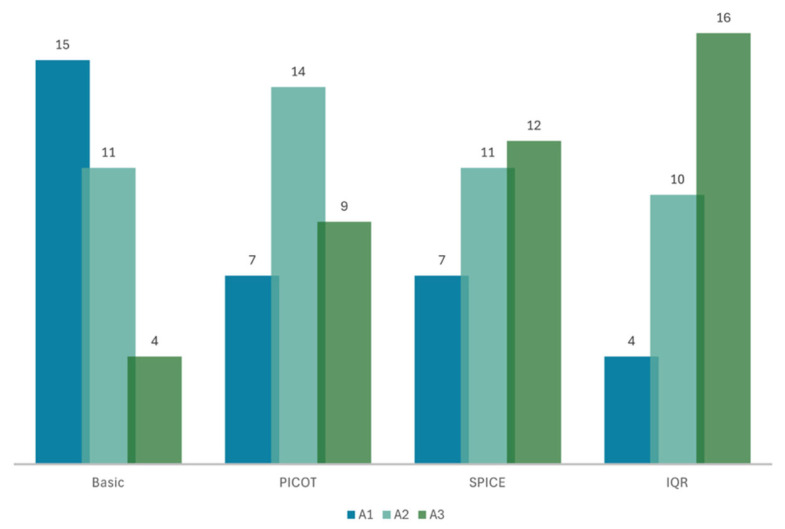
Bar graph showing the models’ accuracy scores on a 3-point Likert scale: “Basic” naïve RAG model; self-query retrieval (SQR) using the PICOT framework; SQR using SPICE; SQR using PICOT with SPICE as a fallback mechanism and iterative query refinement (IQR). A1: accuracy score of 1, A2: accuracy score of 2, A3: accuracy score of 3. There is a distribution shift toward higher accuracy from Basic RAG to PICOT, SPICE, and IQR, with a corresponding drop in low-accuracy ratings.

The model integrating the full SQR pipeline with PICOT, SPICE as a fallback mechanism, and concomitant IQR outperformed the other three models—SQR for PICOT, SQR for SPICE, and naive RAG—achieving a mean accuracy score of 2.40 on a three-point Likert scale with a standard deviation (SD) of ±0.72. This meant that 53% (*n* = 16) of the model’s responses were rated completely accurate, while 87% (*n* = 26) were at least somewhat accurate. Although it proved to be superior to the other models, it was only statistically superior to the naive RAG model, with a *p*-value of less than 0.01. The second-best performing model utilized SQR to restructure prompts into the SPICE framework, achieving a mean score of 2.17 ± 0.79. Specifically, 40% (*n* = 12) of the responses were fully accurate, and 77% (*n* = 23) were at least somewhat accurate. This was also statistically superior to the naive model (*p* < 0.05). The model using SQR to restructure queries into PICOT achieved a mean accuracy score of 2.07 ± 0.74, with 30% (*n* = 9) of the responses being fully accurate and 67% (*n* = 23) at least somewhat accurate. Conversely, the naive model’s accuracy was 1.63 ± 0.72, and only 13% (*n* = 4) of the responses were considered fully accurate. Figure 2 illustrates the accuracy of the models.

Increasing the accuracy from 13% (50%) to 53% (87%) ensures that the information retrieved from the models is safer to use for decision support.

### 3.2. Clinical Relevance

**Figure 3:**
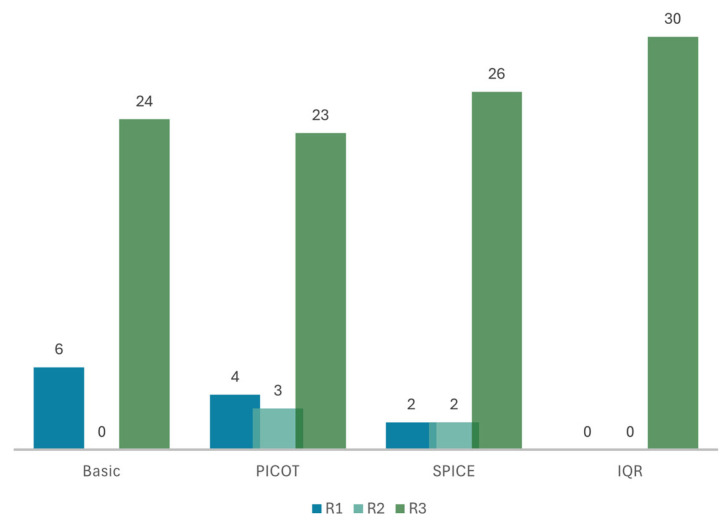
Bar graph showing the models’ relevance scores on a 3-point Likert scale: “Basic” naïve RAG model; self-query retrieval (SQR) using the PICOT framework; SQR using SPICE; SQR using PICOT with SPICE as a fallback mechanism and iterative query refinement (IQR). R1: relevance score of 1, R2: relevance score of 2, R3: relevance score of 3. Structuring queries with PICOT and SPICE reduced low-relevance responses; IQR yielded uniformly high relevance across all prompts.

Similarly to accuracy, the model integrating the full SQR pipeline, with SPICE as a fallback mechanism to PICOT and IQR, achieved the highest relevance score of 100%, outperforming the other models and being significantly superior to the naive RAG model (*p* = 0.03). Restructuring queries into SPICE led to the second most relevant responses, with a mean of 2.80 ± 0.55. Twenty-six were fully relevant (87%), and only two (7%) were irrelevant. The model using the PICOT framework performed very closely to the naive model, with a mean score of 2.63 ± 0.72 vs. 2.60 ± 0.81, respectively. Specifically, 80% (*n* = 24) of the naive model’s responses and 77% (*n* = 23) of the PICOT’s were fully relevant, while 7% (*n* = 4) of the latter and 20% (*n* = 6) of the former were irrelevant. Figure 3 shows the relevance of the models’ responses.

By increasing the relevance of the information retrieved from 80% to 100%, the model can significantly reduce the time spent retrieving clinically useful information.

### 3.3. Information-Retrieval Performance

For information retrieval, the full SQR pipeline with fallback and IQR demonstrated the highest performance. The naive RAG model, utilizing unstructured free-text prompts, achieved a precision of 0.17 and a recall of 0.40, resulting in an F1 score of 0.24. This indicates that fewer than one in five retrieved passages were relevant, and less than half of all relevant items were found. Conversely, integrating the fallback mechanism and IQR increased the precision to 0.53, recall to 1.00, and consequently the F1 score to 0.70. In practical terms, this adaptive SQR technique reduced the proportion of irrelevant results nearly in half (from 83% false positives to 47%), while ensuring that no relevant evidence was missed.

**Figure 4:**
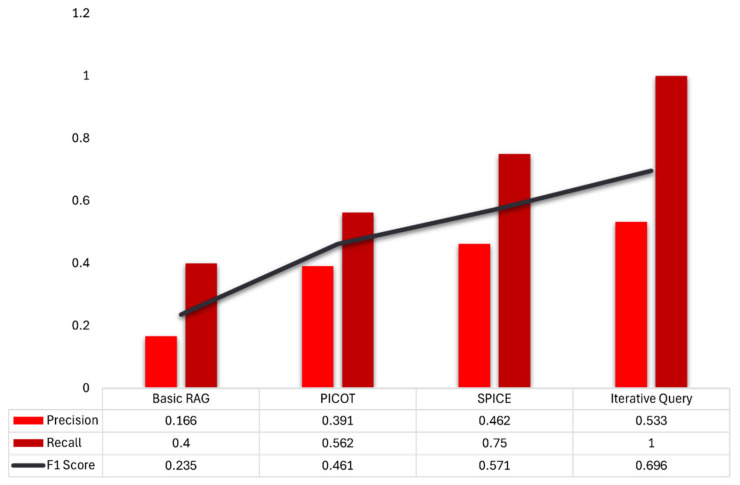
Information Retrieval performance. Bars show precision (light red) and recall (dark red); the line shows F1 (harmonic mean of precision and recall). Structuring queries with PICOT or SPICE mainly increases recall by aligning the prompt with clinically salient elements, reducing missed relevant items. Adding adaptive iterative-query refinement (IQR) further improves precision and recall by iteratively clarifying the query, yielding the highest F1. Metrics are descriptive; definitions appear in Methods.

On the other hand, structuring prompts with the PICOT framework doubled the precision to 0.39 and improved recall to 0.56, resulting in an F1 score of 0.46. SPICE-formatted prompts further increased these metrics to 0.46, 0.75, and 0.57, respectively. These results demonstrate that as prompts become more structured, and especially when refined iteratively, the RAG-LLM provides more accurate and comprehensive clinical guidance. Figure 4 provides a graphic comparison of these results.

### 3.4. Self-Query Retrieval and Iterative-Query Refinement

To illustrate how the IQR improves original user queries, Table 1 presents the different restructured prompts, along with their worst- and best-scoring iterations and their final retrieved responses.

## 4. Discussion

This study evaluated a self-query retrieval (SQR) pipeline with iterative-query refinement (IQR) to address the semantic gap that impedes naive RAG-LLM performance in a clinical decision-support setting. By restructuring patient queries into PICOT and reverting to SPICE when needed, and allowing the model to critique and refine its own prompts, we demonstrated marked improvements in accuracy, relevance, and information-retrieval (IR) metrics for post-rhinoplasty questions. In particular, the full SQR  +  IQR pipeline achieved a mean accuracy of 2.40 ± 0.72, a perfect recall of 1.00, and an F1 score of 0.70, substantially outperforming both naive RAG (accuracy 1.63 ± 0.72, recall 0.40, F1 0.24) and partial SQR implementations. These results suggest that embedding structured, metadata-aware prompts and iteratively refining them can substantially enhance retrieval and potentially reduce the likelihood of clinically unsafe or irrelevant recommendations.

### 4.1. Interpretation of Findings

Consistent with prior findings that prompt quality strongly influences LLM outputs, the introduction of SQR alone, whether via PICOT or SPICE, already doubled the precision (from 0.17 to 0.39–0.46) and more than doubled the recall (from 0.40 to 0.56–0.75) [54,56,57,58,59]. In our pipeline, forcing the LLM to decompose the user’s question into clinically meaningful components ensured that retrieval focused on the most salient metadata, such as procedure type, postoperative day, and target outcomes, thus narrowing the candidate document set to highly relevant passages. In contrast, Naive RAG treats the entire free-text query as a single embedding, making it prone to retrieving semantically similar but clinically irrelevant content [60].

Iterative refinement further improved performance, with the full pipeline achieving perfect recall (1.00) while reducing false positives from 83% in the naive RAG to 47%. This result aligns with findings from Ma et al. and Peimani et al., who demonstrated that rephrasing or rewriting queries prior to retrieval closes the semantic gap and yields higher information retrieval (IR) quality. Specifically, Ma and colleagues demonstrated that introducing a rewriting step before retrieval outperformed standard RAG on open and multiple-choice questions, while Peimani et al. observed that adding domain-specific terms improved top similarity scores. In our context, the IQR loop mimics these strategies by having the LLM self-critique and refine, rather than relying on a single static rewritten query [44,45]. Importantly, by the tenth iteration, both our composite retrieval score and answer phrasing plateaued. This finding is consistent with prior convergence analyses, which validate the ten-step cap as a practical compromise between performance gains and computational cost [46,52].

Notably, the IQR-augmented pipeline also achieved 100% fully relevant outputs, compared to 80% for naive RAG and 87% for SQR using SPICE, reinforcing the notion that context and prompt specificity substantially influence outcome relevance [57]. Koopman and Zuccon showed that in healthcare prompts, simply injecting evidence or turning them to their negative can reduce LLM performance from 80% to 63%, illustrating that even minor wording shifts can have a significant impact on accuracy [61]. Our structured prompts resolve this by standardizing the query format, thereby protecting retrieval from variations in idiosyncratic phrasing.

### 4.2. Comparison with Current Research

Previous work on prompt engineering has established that model responses are highly sensitive to the wording and structure of the initial prompt, especially in healthcare domains [54,56,57,58,59,61]. Chen et al. introduced a model-adaptive prompt optimizer (MAPO) that tailors prompts to each LLM, demonstrating that even well-crafted prompts benefit from model-specific tuning [54]. Our pipeline follows this logic by allowing the model to rephrase its own query, thereby effectively adapting prompts in real-time to the LLM in use, without requiring separate parameter tuning.

Efforts to bridge the semantic gap in RAG include multi-query rewriting [51], query-enhanced retrieval [57], and generating question sets from knowledge databases [46]. Kostric and Balog demonstrated that rewriting conversational queries enhanced retrieval in passage retrieval models [51], whereas Yang et al.’s Query-Based RAG aligned a comprehensive question set with user input to better match documents [46]. These approaches resonate with our SQR strategy of extracting metadata fields and reformulating queries accordingly. Unlike static rewriting, our IQR mechanism continuously updates the prompt based on intermediate retrieval results, therefore combining the benefits of meta-query extraction with dynamic correction of residual errors [44,45].

Recent clinical implementations of RAG-LLMs have underscored the promise of retrieval to reduce hallucinations and improve answer fidelity [40]. In nephrology, Miao et al. demonstrated that a RAG-LLM could outperform a fine-tuned model in suggesting evidence-based interventions; similarly [31], Ong and colleagues showed that RAG-enhanced LLMs improved guideline adherence in oncology [32]. However, they join other authors’ conclusions, recognizing that optimizing RAG architectures still remains a challenge [28]. Our results extend this body of work by demonstrating that structured query decomposition and adaptive refinement can further enhance retrieval gains in a specialized setting such as post-rhinoplasty care.

### 4.3. Impact on the Medical Practice

By improving precision (0.53 vs. 0.17) and recall (1.00 vs. 0.40) compared to naive RAG, the SQR  +  IQR pipeline directly addresses physician concerns about reliability and accountability in AI-driven decision support [23,62]. In busy clinical settings, a rapid and trustworthy answer is essential; poorly phrased prompts or inadequate retrieval can mislead clinicians and compromise patient safety. In rhinoplasty care, where postoperative instructions are well-defined, mis-retrieval carries a non-negligible but lower risk, enabling us to demonstrate safety and efficacy in a controlled domain before scaling to higher-risk specialties. Reducing false positives by nearly half not only minimizes noise but also saves physicians time, as they do not need to strain while reading irrelevant passages, aligning with our goal of decreasing cognitive burden and allowing physicians to focus on patient interaction [54].

The implications extend beyond rhinoplasty. A robust SQR  +  IQR pipeline could support any clinical domain with standardized care pathways (e.g., postoperative protocols in orthopedics or chronic disease management), while still being useful in more unpredictable domains due to its RAG nature and intermediate refinement architecture, such as clinical or surgical decision support. By ensuring that no relevant evidence is missed (e.g., perfect recall) and by maximizing precision, this approach can mitigate the risk of AI hallucinations and enhance clinician trust. In turn, more confident adoption of RAG-LLMs may accelerate integration of AI assistance into routine workflows, potentially improving diagnostic accuracy, reducing preventable errors, and streamlining documentation [4,9,10].

### 4.4. Limitations

Despite the promising results, several limitations need to be addressed. First, our evaluation was limited to 30 rhinoplasty queries, a number sufficient for a proof-of-concept but small relative to the heterogeneity of clinical questions. This sample may not capture the full spectrum of postoperative scenarios, such as late complications or complex revision cases, potentially biasing performance estimates. Second, our knowledge corpus consisted exclusively of authoritative rhinoplasty sources, whereas in real-world deployment, the retrieval system would encounter a far larger and more heterogeneous document set, including mixed-quality web articles, institutional notes, and potentially, dynamic data from Electronic Health Records (EHR). The controlled corpus likely amplified retrieval performance; scaling to an open corpus could introduce noise, requiring further tuning of composite weights or more aggressive filtering. Future studies will focus on enriching our knowledge corpus to cover the broader spectrum of clinical decision-making, such as including information on further diseases and procedures. Additionally, testing will not only focus on medical accuracy and relevance but also real-time application and physician perception of actual usability and workflow efficiency.

Third, we fixed the composite-score weights (0.5 semantic, 0.3 lexical, 0.2 length) based on ablation tests within this domain. However, these weights may not generalize to other specialties where lexical cues or chunk lengths behave differently. A dynamic, data-driven weighting strategy might improve generalizability. Fourth, while our ten-iteration IQR cap balanced performance and cost in preliminary tests, more complex queries or larger corpora could require more iterations to converge; conversely, some queries might converge in fewer passes, suggesting that adaptive stopping criteria (e.g., monitoring plateau rates) could yield further efficiency gains without sacrificing accuracy. Future studies will assess the feasibility of real-time application of the framework and determine its impact on clinical workflow efficiency and the quality time spent with the patient.

Fifth, our results rely on a specific LLM and a chosen embedding model. Given the known variability in embedding fidelity and LLM architectures, performance could differ if alternative models are used [60,63]. Future evaluations should compare multiple embedding and LLM combinations to identify optimal pairings. Finally, although our focus on rhinoplasty minimized clinical risk, it also limits immediate generalizability to high-stakes domains, such as oncology treatment planning or ICU management, where erroneous guidance could have severe immediate consequences. Formal prospective studies in those areas will be necessary before clinical deployment.

## 5. Conclusions

Our investigation demonstrates that combining self-query retrieval with iterative refinement substantially improves RAG-LLM performance for clinical decision support, as evidenced in the postoperative rhinoplasty setting. By leveraging structured metadata via PICOT/SPICE and enabling the model to refine its own queries, we closed the semantic gap that typically hinders naive RAG architectures. Although our work is confined to a well-defined, low-risk domain, it lays the groundwork for more ambitious deployments across a range of specialties. By reducing clinician workload and delivering highly accurate, evidence-grounded answers, SQR  +  IQR pipelines have the potential to transform how physicians access and apply knowledge, ultimately improving patient care and safety in an increasingly data-driven healthcare environment.

## 6. Future Directions

Building on these findings, further research is warranted. Replication in other procedural domains would test the pipeline’s adaptability. Each specialty has its own set of metadata attributes (e.g., implant type, fusion level, comorbidity scales); therefore, customizing structured schemas beyond PICOT/SPICE, such as ONCO-PI(E) in oncology or RADS-Q in radiology, could enhance performance. Moreover, integration with simulated real-time EHR feeds and patient-specific data (e.g., lab values, medication lists) could personalize retrieval. For instance, post-renal transplant queries might automatically incorporate the patient’s latest creatinine level, enabling the system to surface immunosuppression guidelines tailored to kidney function.

Additionally, a user study with practicing clinicians could evaluate downstream effects on workflow efficiency, decision confidence, and patient outcomes. Metrics such as time to answer, number of clinician corrections, and patient satisfaction would provide critical validation beyond IR metrics. Exploring dynamic composite-score weighting, potentially via reinforcement learning or meta-optimization, could further optimize retrieval for each query type, addressing the limitation of fixed weights. Fifth, expanding the IQR mechanism to consider multimodal feedback, including clinician ratings of initial answers and click-through data rather than solely LLM self-critique, could improve refinement accuracy and reduce convergence time.

Finally, although our proposed framework addresses a crucial step in the RAG architecture, further research is needed to explore how to improve various components in the pipeline. Identifying optimal chunking methods and retrieval strategies could further enhance the efficiency of RAG-LLMs and improve patient safety.

## Figures and Tables

**Table 1:** Demonstration of the SQR restructured prompts and iterative refinement. Question 1 shows the model using the PICOT framework and iterative query refinement to increase the composite context score by 16%. Question 2 illustrates the self-query retrieval into the SPICE schema and its iterative refinement, resulting in a 34% enhancement. Question 3 demonstrates the fallback mechanism once the model encounters two missing components of the PICOT framework and the subsequent iterative refinement.

**1. Original query:** “I had aesthetic rhinoplasty with an open technique three days ago. What should I apply to the incision?”		
**Worst scoring iteration:** Population: Patients three days post-open rhinoplasty Intervention: Topical applications to the incision site Comparison: No treatment/standard postoperative care (this is implied as the query asks what can be applied, suggesting they are not currently using anything beyond standard care) or surgeon-recommended treatment (as the query specifically asks about surgeon recommendations, comparing other options to the surgeon’s advice is relevant) Outcome: Improved healing and minimized scarring Time: Three days post-surgery **Refined Query = Three days after open rhinoplasty, what can I apply to my incision to aid healing and minimize scarring? Is there anything my surgeon specifically recommended I use?** Score = 0.4847764803303612	**Best scoring iteration:** Population: Patients three days post-open rhinoplasty Intervention: Topical application of ointments/creams or other treatments to the surgical incision Comparison: Keeping the incision dry, or alternative surgeon-prescribed treatments (implicitly including standard postoperative care) Outcome: Improved healing and minimized scarring Time: Three days post-operatively **Refined Query = Three days after open rhinoplasty, what can I apply to my incision to aid healing and minimize scarring? Should I be using an ointment, keeping it dry, or something else prescribed by my surgeon?** Score = 0.5628299422123852	**Final Response:** After an open rhinoplasty, patients should do the following to the incision three days after the procedure: Gently clean the visible sutures in the columella (if present) and nasal vestibule to prevent crusting. To clean the sutures, use a solution of half-strength hydrogen peroxide (the patient may mix equal amounts of water and hydrogen peroxide) applied with a cotton-tipped applicator. Instruct patients before surgery that doing this consistently will make suture removal faster and less painful, and they will almost always comply. After cleaning the sutures, apply a thin coating of antibacterial ointment to the sutures. Clean in this way two to three times per day to prevent crust accumulation in most cases. Keep the cast dry if one has been applied.
**2. Original query:** “I had aesthetic rhinoplasty with an open technique three days ago. When can I wear my glasses?”		
**Worst scoring iteration:** Setting: Postoperative care, outpatient. Population: Patients who have undergone open rhinoplasty. Intervention: Wearing glasses resting on the bridge of the nose. Comparator: Not wearing glasses or wearing glasses without a bridge contact (e.g., taped to forehead). Evaluation: Time until safe resumption of wearing glasses on the bridge of the nose post-rhinoplasty (i.e., without risk of complications such as displacement of nasal bones or cartilage). **Refined Query = I had open rhinoplasty three days ago. When can I safely rest glasses on the bridge of my nose again?** Score = 0.3924790531410891	**Best scoring iteration:** Setting: Postoperative (3 days post-op) Population: Adult patient who underwent open rhinoplasty Intervention: Wearing glasses Comparator: Not wearing glasses/Alternative methods of vision correction (e.g., contact lenses) Evaluation: Time until safe resumption of glasses wearing; Strategies to minimize nasal pressure **Refined Query = I had open rhinoplasty 3 days ago. How long should I wait before wearing glasses, and what can I do to minimize pressure on my nose in the meantime?** Score = 0.5274241349913857	**Final Response:** 6 weeks: This is the general timeframe most surgeons recommend waiting before wearing glasses again after open rhinoplasty. This allows the nose adequate time to heal and minimize the risk of pressure or displacement on the newly restructured tissues.
**3. Original query:** “I had aesthetic rhinoplasty with an open technique three days ago. I have a persistent headache. What should I do?”		
**Fallback and worst scoring iteration:** Population: Patients who have undergone open aesthetic rhinoplasty Intervention: N/A (The intervention is the rhinoplasty itself, and we are looking at a consequence, not comparing interventions) Comparison: N/A (Not comparing different interventions or a lack of intervention) Outcome: Persistent headache Time: Three days postoperative **→ Missing components: 2** **↪ Iteration 0 PICOT failed/incomplete: Two of the PICOT components are missing.** **→ Switching to SPICE fallback.** Setting:Postoperative (3 days post-op) Population: Patients who have undergone open rhinoplasty Intervention: Experiencing persistent headache Comparator: (Implicit) Not experiencing a headache/Normal postoperative course Evaluation: Normality of the headache, Management/treatment options for the headache **Refined Query = “Three days after open rhinoplasty, I’m experiencing a persistent headache. Is this normal, and what should I do about it?”** Score = 0.429184089201252	**Best scoring iteration:** Setting: Postoperative (3 days post-op) open rhinoplasty Population: Patients undergoing open rhinoplasty Intervention: Headache management strategies Comparator: N/A (The question is about managing an existing headache, not comparing interventions.) Evaluation: Headache resolution/reduction; Normality of persistent headache post-op. (Implicitly, the query also seeks to evaluate whether contacting the surgeon is necessary). **Refined Query = “I had open rhinoplasty three days ago and I have a persistent headache. Is this normal, and what can I do to alleviate it? Should I contact my surgeon?”** Score = 0.526509184295481	**Final Response:** I understand you are concerned about sun exposure after your open aesthetic rhinoplasty a week ago. Based on the information in the provided context, here is what I can tell you: Excessive heat and sun exposure to the nose should be avoided for 3 months after the operation This includes sunbathing, solarium treatments, and even prolonged exposure to direct sunlight. It is important to protect your nose while outdoors. Wear a wide-brimmed hat and apply sunscreen with an SPF of 30 or higher to the exposed areas of your face, including your nose.

## Data Availability

The datasets generated and/or analyzed during the current study are not publicly available but are available from the corresponding author on reasonable request.
